# Current plastics pollution threats due to COVID-19 and its possible mitigation techniques: a waste-to-energy conversion via Pyrolysis

**DOI:** 10.1186/s40068-020-00217-x

**Published:** 2021-01-20

**Authors:** Tadele Assefa Aragaw, Bassazin Ayalew Mekonnen

**Affiliations:** 1 Faculty of Chemical and Food Engineering, Bahir Dar Institute of Technology-Bahir Dar University, Bahir Dar, Ethiopia; 2 Bahir Dar Energy Center, Bahir Dar Institute of Technology-Bahir Dar University, Bahir Dar, Ethiopia

**Keywords:** PPE plastics, COVID-19, Characterization, Pyrolysis, Fuel

## Abstract

**Background:**

The extensive use and production of PPE, and disposal in the COVID-19 pandemic increases the plastic wastes arise environmental threats. Roughly, 129 billion face masks and 65 billion plastic gloves every month are used and disposed of on the globe. The study aims to identify the polymer type of face masks and gloves and sustainable plastic waste management options.

**Results:**

The identification of polymers, which can help for fuel conversion alternatives, was confirmed by FTIR and TGA/DTA analysis and confirms that the polymeric categories fit for the intended purpose. Moreover, the handling technique for upcycling and the environmental impacts of the medical face mask and glove were discussed. The FTIR result revealed that face masks and gloves are polypropylene and PVC thermoplastic polymer, respectively and they can be easily transformed to fuel energy via pyrolysis. The endothermic peaks around 431 ℃ for medical glove and 175 ℃ for surgical is observed tells that the melting point of the PVC and polypropylene of plastic polymers, respectively. The pyrolysis of the face mask and glove was carried out in a closed reactor at 400 ℃ for 1 h. Conferring to lab-scale processes, liquid, and wax fuel rate of 75%, char of 10%, and the rest non-condensable gases were estimated at the end.

**Conclusions:**

It can be concluded that the medical plastics can be recycled into oil due to their thermoplastics nature having high oil content and the waste to energy conversion can potentially reduce the volume of PPE plastic wastes.
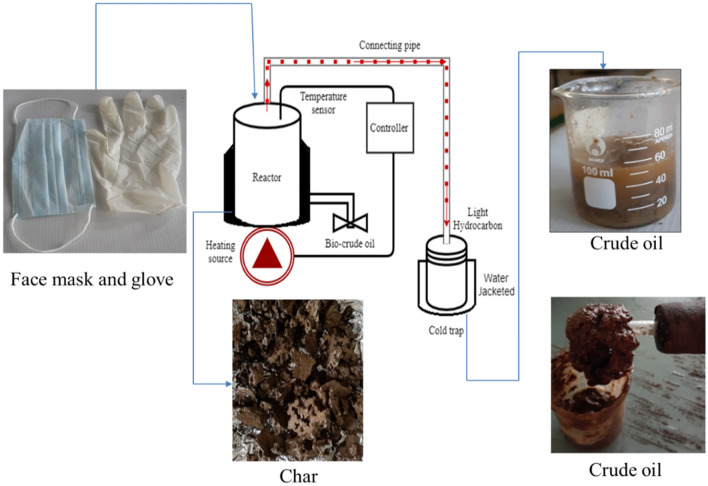

Highlights

- Polymer type identification of the medical face mask and glove are carried out.
- PPE wastes generated during pandemic affect waste management.
- The COVID-19 pandemic surges carbon footprint to the environment.
- PPE waste handling methods before reusing were highlighted.
- Fuel recovery through pyrolysis for sustainable waste management was stated.
- The environmental impacts of PPE wastes during the COVID-19 scenario were discussed.

## Introduction

Global communities are becoming highly dependent on utilizing plastic polymers since commercial production began in about 1950. These days, plastics are become global demand due to simplicity, flexibility, low density, and low production costs. The worldwide plastic production in the globe has been projected at approximately 300 million tons per year and is endlessly increasing every single year (Miandad et al. 2016; Ratnasari et al. 2017). Accordingly, plastic-made material usage and plastic waste production are rising at an alarming rate and its short service life accelerates the plastic waste generation every day (Miandad et al. 2019). Nowadays, the COVID pandemic era has caused an increase in the amount of plastic waste and medical waste, personal protective equipment (PPE), generation worldwide aplastic pollution in the environment (De-la-Torre and Aragaw 2021). Consequently, billion times the tones of extra plastic products have been producing in the globe for the protection of COVID-19 transmission. For instance, human beings are using and disposing of roughly 129 billion face masks and 65 billion plastic gloves every single month globally. According to (Mahbubani 2020) reported in March 2020, 89 million face masks, 76 million hands-on gloves, and 1.6 million protective goggles are required monthly worldwide. So, around 200 Billion facemasks and gloves are going into the environment every month. As a result of a one-time use of these surgical masks followed by their disposal during this COVID-19 augments the burden of polymer on the globe (Jain et al. 2020). Therefore, the use and mismanagement of medical waste by the COVID-19 pandemic is contributing to the increasing plastic contamination. In the meantime, the persistence of PPE deposes of the COVID-19 pandemic will probably be a common plastic debris item that existed in the surroundings for decades. Moreover, single-use disposable plastics have been recognized as a major cause of micro-plastic litter in the environment (Schnurr et al. 2018). Particularly, recent studies validate that the medical face masks are potential sources for micro-plastic pollution in the water systems, and become an environmental threat in the COVID-19 scenario (Aragaw 2020). Thus, the current pandemic caused an increase in the amount of plastic and medical waste generation. Due to this reason, an abundant amount of non-biodegradable polymers including PPE is now disseminated widely around the world in different forms and applications. As a result, an enormous quantity of macro-plastics and their fragmented particles are disposed to the surroundings as well as the risk of the environmental pollution load is predicted as doubling and more than doubling year to year. Undeniably, this PPE plastic management becomes an exceptional concern due to the risk of biohazards nature. Unfortunately, a consistent PPE waste management policy has not been achieved due to the high bio-hazardous potential of the waste (Bdour et al. 2007). In addition, the heterogeneous composition of PPE waste makes it challenging for proper separation and recycling (Wu et al. 2019) (Anuar Sharuddin et al. 2016). Furthermore, recycling only delays final disposal and the plastics obtained have limited economic and technological characteristics. Another option of landfills of plastic offers high resistance to degradation by microorganisms at room temperature and therefore persists for long periods(Jain et al. 2020; Ma et al. 2017). Consequently, conventional landfill practices are losing their acceptance due to harmful environmental effects and the low degradation nature of plastic polymers (You et al. 2020). In these perspectives, thermal destruction treatment offers the utmost reliable disposal route for PPE (Al-Salem et al. 2017) (Qin et al. 2018). Among the thermal degradation processes, incineration of PPE is the usual practice for the disposal of PPE (Geyer et al. 2017), but it lacks the technical merit of air pollution controls (Makarichi et al. 2018). Similar to the public bio-medical wastes, the COVID-waste has been incinerated at a temperature above 1100 °C (Ilyas et al. 2020). Thus, several toxins are generated in place incineration like furan, and dioxins cause severe health problems (Aragaw 2020) (Ilyas et al. 2020). In addition, incineration along with flue-gas purification also required additional costs and load to the worker. Hence, the operation of an incineration facility with flue-gas cleaning for a small quantity of PPE wastes is not feasible and applicable for handling alternative technologies (Ilyas et al. 2020). Besides, the incineration of chlorine-containing plastics such as PVC generates toxic chemicals that cause cancer, if not done at the right temperature leads to adverse effects on the ecosystem (Klemeš et al. 2020).

Accordingly, finding a reliable disposal platform for PPE beyond incineration and landfilling has great importance (Qin et al. 2018). In the future, pyrolysis offers an effective means for reusing via recovering energy eradicate waste management problems (Burra and Gupta 2018). Because pyrolysis has superior merit towards pollution and a decrease in the carbon footprint of plastic products compared to other thermal treatments(Al-Salem et al. 2017). Moreover, it does not need earlier separation of dissimilar waste plastics; hence a mix of plastics can also be converted into crude bio-oil, which is probably reprocessed for the generation of energy based on end-users and commercial applications. To this end, the present study aims to reduce the use of plastics (SUPs) arising from the surge in disposal PPE usage with a particular focus on pyrolysis as a means of the conversion of PPE (facemask and surgical glove) constituent plastics to fuel. The experimental characterization of polymer type and analysis of waste recovery potential via pyrolysis was done on a lab-scale basis including highlighting the handling and pretreatment options of the medical wastes. The key experimental results; polymer type identification, waste conversion rate, and future perspectives were reported for waste management recommendations for future implications.

## Materials and methods

### PPE waste handling and preparation for upcycling

The specific personal protectives components for healthcare workers (HCW) comprise gloves, gowns, shoe covers, head covers, masks, respirators, eye protection, face shields, and goggles. Each personal protective components either alone or in combination have a specific application for contamination prevention. For instance, gloves and grows are worn to protect directly handling potentially infectious materials or contaminated surfaces as part of standard precaution or contact precaution. While shoes and head cover are worn for protection of the likely exposure to a polluted environment as part of full barrier safety measures against airborne organisms, or contact with a contaminated environment is anticipated. Another PPE infectious control equipment is mask and respirators consists of surgical masks, disposable respirator (N95), powered air-purifying respirator (PAPR), and Self-contained breathing apparatus (SCBA) respirators. All these protective types of equipment are worn for protection of mouth and nose from splattered body fluids, microorganisms (bacteria and viruses), and respirators filter the air before inhaling it. Furthermore, goggle and face shield personal protectives provides protection of eyes from splatters and splatter protection to facial skin, eyes, nose, and mouth respectively as a standard precaution for medical applications(Minnesota Department of Health 2020).

All the stated personal protectives are disposable medical wastes that have high risks of infections with pathogens, viruses, and bacteria. Principally, all COVID-waste is recognized as hazardous biomedical wastes and required disinfection before use (Ilyas et al. 2020). As a result, the effective management and safe handling practices of this waste are paramount. Ilyas et al. proposed the COVID-19 hospital and biomedical wastes treatment technique (incineration, thermal, and chemical treatment) for disinfection and reprocessing of medical wastes (Ilyas et al. 2020). Among the decontamination method of PPE, incineration, and alternative thermal disinfection are intended for waste volume reduction rather than reprocessing to different products (Ilyas et al. 2020). Despite the rigorousness of sterilization modalities, conventional sterilization technologies also degraded the quality of PPE. Thus, the one-time-use medical plastics are not suitable for re-using later the post-treatment. In addition, high-level disinfection is found to change characteristic personal protective components (Rowan and Laffey 2020a). Hence, the above-described handling technologies are suitable for the disinfection of contaminates for safe disposal only. However, the chemical (chlorine and non-chlorine), vaporized hydrogen peroxide (vH_2_O_2_), and UV-radiation (typically, Claranor, France, and (Nanoclave cabinet, Ireland) effectively disinfects the virus spores without reducing the volume of disposable medical waste (Rowan and Laffey 2020b) (Barcelo 2020). Therefore, these treatment methods provide an opportunity for re-use with no threat to change in intrinsic characteristics and quantity of plastic waste volume. With this regard, chemical, vH_2_O_2_, and/or UV radiation treatment technology could be deployed for proper handling for later reusing of disposable plastic wastes. But in the present study, single-use face masks and gloves were purchased from a pharmacy store in Bahir Dar-Ethiopia for lab-scale analysis. Then samples were chopped into small pieces. However, in the real case, medical wastes pretreatment should be implemented before reprocessing to other products using proper disinfection techniques for specific repurposing of it.

### Characterization of medical waste plastics

PPE recovering must consider material composition, the functionality of post-treatment, along with appropriate fit for purpose. Otherwise, it is important to follow the PPE manufacturer governing guidance of the detailed features of the PPE. However, PPE is thermally sensitive while it may not found within the manufacturer’s design quality proposed for reprocessing (Rowan and Laffey 2020b). So, before upcycling of the disposable PPE, its polymer type identification, thermal degradation property should be identified using Fourier-transform infrared spectroscopy (FTIR) and Thermo-gravimetric analysis.

#### FTIR analysis

The chemical structure determination is an important technique that can give us the basic information to identify the polymeric type. The face mask and glove were chopped separately with the scissor and grounded with mortar and pestle. The pellets were prepared with reagent grade potassium bromide (KBr) powder with mechanical pressing. The spectral analysis was analyzed using a JASCO-6600 spectrometer with a scanning range of 400–4000 cm^− 1^.

#### Thermo‐gravimetric analysis

The thermogravimetry (TGA) instrument, BJ HENVEN (ATAT 2012) was used for the differential thermo-gravimetric curve determination of medical face masks and glove plastic polymers. Firstly, the medical face mask and gloves were chopped with a small size to be appropriate for the instrument crucible. The 6.5 mg mass chopped medical face mask and glove were separately weighed with analytical balance (KE-ABJ80-4NM), and are put on the ceramic crucible. As a blank, the empty crucible was used aside from the sample crucible. The differential thermalgravimetric analysis (DTA) was heated from ambient temperature to 600 °C with a heating rate of 15 °C/min, a TGA range of 100, and a sampling cycle of 1500. The time for complete thermal analysis was required 40 min.

### Pyrolysis and experimental setup

**Fig. 1:**
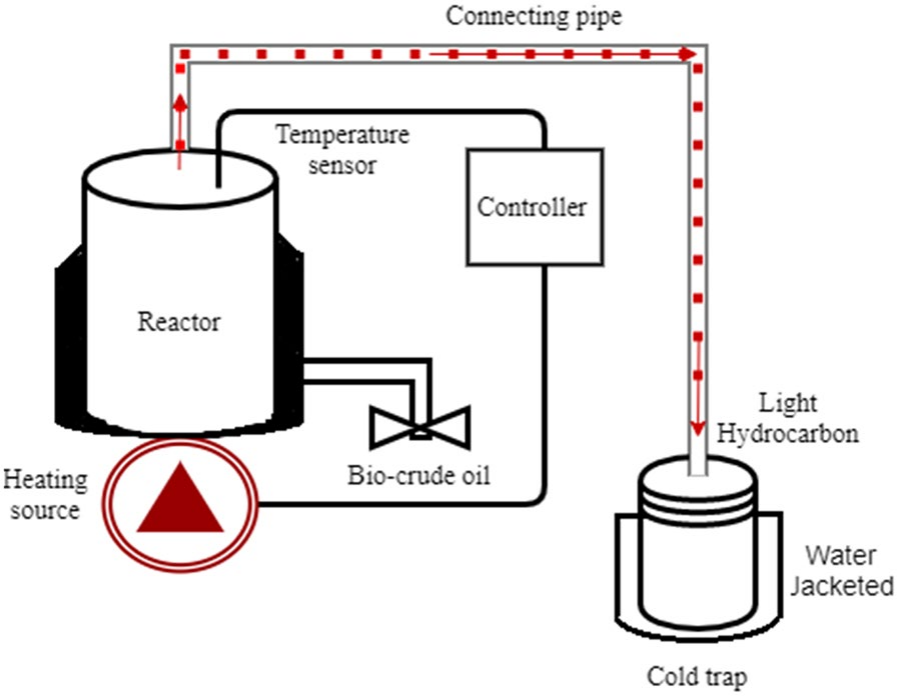
A benchtop pyrolysis setup for fuel production

The pyrolysis experimental setup is shown in Fig. 1. It comprises an insulated 10 cm in diameter and 13.5 cm height steel closed can reactor, a 10 mm flexible connection pipe, and a water-jacketed receiving flask. The reactor vessel and a cold trap are connected with a flexible metallic hose. Before the experiment, raw materials (fresh face masks and surgical gloves) are purchased from local pharmacies and chopped into pieces. Then, 300 gm of face mask and surgical glove each weighing 150 gm was feed to the reactor. The reactor contents are then sealed and placed in the heating mantle. The thermal treatment was performed at 400 °C for 1 h in the closed air system with no vacuum process applied during this thermal cracking process. Finally, the pyrolysis product was sent to the water jacket cold trap and allowed to cool to room temperature.

Hypothetically, waste plastic pyrolysis was taken place at 350–500 °C for two h. Upon heating, the plastics are subjected to depolymerization, pyrolysis, thermal cracking to obtain pyrolyzed fuel oil. Heat is applied from 100 °C at the start to initiate melting the plastics, the melted waste plastic turn into liquid slurry form when the temperature is increased gradually. When the temperature is raised to 270° C liquid slurry turns into vapor for the first 25 min and the vapor is then passed through a connection tube to the condenser unit. Thermal cracking continues for the next 35 min, while the temperature was kept at 400 °C. During this thermal cracking process, some light gases are produced and the condensable pyrolyzed gases were continuously swept out from the reactor. In the end, the entire heated vapors coming out of the reactor are condensed on a cool water-jacketed receiver for cooling of the vapor. The gaseous hydrocarbons at a temperature of about 400 °C are condensed to about room temperature 30–35 °C by direct contact to a water-jacketed cold trap. However, heating was limited for up to 1 h time, because of difficulty in maintaining a gas seal resulting in gas leakage in this small-scale experimental setup. At the end of the experiment, the reactor and cold trap content are weighed and measured for pyrolysate components analysis of recovered crude liquid oil and remaining char from the specified sample of disposable face mask and gloves.

In large scale applications, PPE used for healthcare from health and quarantine centers will be collected along with rigorous infection control practices and decontamination methods. After precaution handling, the SUP waste then goes to the central thermal treatment plant for resource recovery. Thus, the wastes can be converted to value-added fuels similar to other solid waste and plastic pyrolysis. Moreover, evidence has shown that a substantial increase in the use/consumption of SUPs and PPE, along with the increment in medical waste inherent to the pandemic, is likely leading to an excess single-use plastic waste generation that potentially used for advanced resource recovery (Prata et al. 2020). The large-scale conversion of PPE and SUP in a central pyrolysis plant will alleviate a sudden surge in demand for consumption of plastic products by the general public, healthcare workers, and service workers imposed by the pandemic and the plastic waste trade stress. Consequently, recovered resources from PPE and SUPs introduced the public impression of medical plastic waste from pain to advance through precise matching the socioeconomic, energy, and environmental demands.

## Results and discussion

### FTIR and polymer type identification

**Fig. 2:**
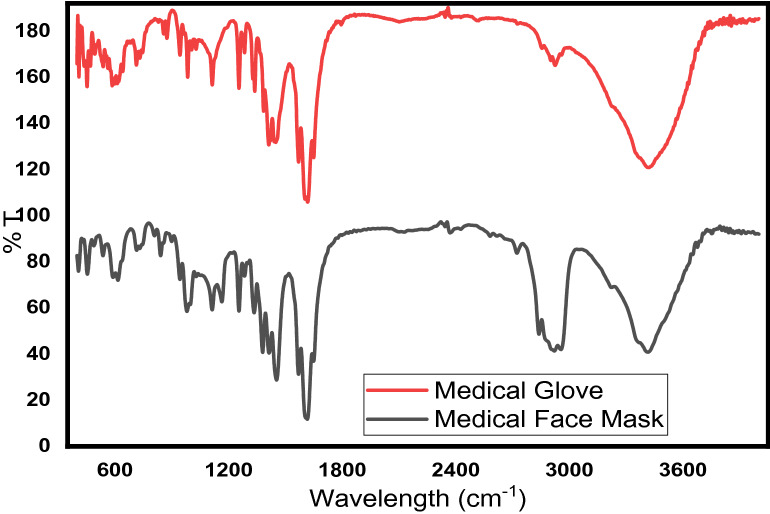
Infrared Fourier transform spectrum absorption bands of medical face mask and glove

Fourier-transform infrared spectroscopy (FTIR) spectral analysis of medical face mask and glove were shown in Fig. 2. The peak at 611 cm^− 1^ for the surgical face mask is corresponding the = C-CH_3_ in aliphatic and phenyl in romantic vibration the monomeric polypropylene polymer (Zhang et al. 2013). The sharp and clear peak at 2900 cm^− 1^ for both medical face mask and glove attributes for the C-H vibration (Nam et al. 2016). The peaks around 1113 cm^− 1^ are assignments for the C-O stretc.hing in the polypropylene constitute of face mask and nitrile rubber made up of medical gloves. The peaks at 1454 and 1380 cm^− 1^ for both face mask and glove are the assignments of the symmetry deformation of –CH_2_– on the aliphatic hydrocarbons (Zhang et al. 2013). Diao et al. reported that the non-woven, and pure polypropylene fabrics of IR spectral were similar to the present study (Diao et al. 2017). The sharp and low band peaks at 1619 and 1545 cm^− 1^ for both glove and face mask is assigned for the carbonyl (C = O) stretc.hing vibration of the primary amide for the polypropylene face mask and the nitriles functional group (− C ≡ N) stretc.hing for nitrile rubber gloves, respectively (Chen and Sun 2005). The broadband and clear peaks for the two polymers, PVC and polypropylene, around 3414 cm^− 1^ attributes the aliphatic or aromatic O-H stretc.hing. The presence of the nitrile group in the rubber type of polymer in the glove plastic was identified can be confirmed at the peak of 2340 cm^− 1^, and the peak at 597 cm^− 1^ is a representative of C-Cl stretc.hing vibrations (Nirmal Ghosh et al. 2017). In general, spectral vibration and stretc.hing can provide evidence face masks and gloves are made up of PVC and polypropylenes which are thermoplastic types of polymer that suggest that it can be used for fuel energy through the pyrolysis process.

### Thermogravimetric analysis and thermal properties

**Fig. 3:**
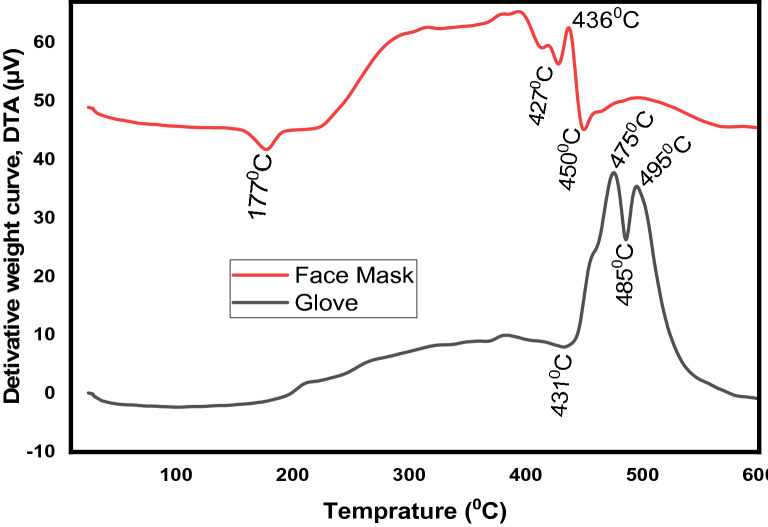
differential thermogravimetric curves of medical face mask and hand-on gloves

The derivatives of the thermogravimetric curve for the medical face mask and hands-on gloves as a function of temperature were computed as shown in Fig. 3, which is commonly used to represent the weight loss of TGA thermograms. The differential thermogravimetric (DTA) curve patterns can give the quantified records on the maximum temperature of the exothermic and endothermic signals. Besides, the DTA pattern, quantified the part of the exothermic and endodermic part yielded the enthalpies associated with the thermal phenomena of the materials. A small endothermic around 210 ℃ of the medical glove is the melting point stage of the PVC polymers which officially know that in the range of 100 to 260 ℃ depending on the manufacturer additives (Causin et al. 2009). The endothermic peak around 431 ℃ for glove plastic polymer is observed. This peak is due to the decompositions of poly-cis-1,4-isoprene organics which contained doing the formulations of PVC glove (de Oliveira et al. 2006). The derivative of the curve concerning temperature originated by the thermo-oxidative degradation and informative with the corresponding thermogravimetric mass loss. An exothermic peak at 475 and 495 ℃ and an endothermic peak at 485 ℃ for the glove ascribed to the combustion of the main polyisoprene chains and the organic additives (Agarwal et al. 2011). The endothermic peak at 175 ℃ of the medical hand-on glove is the melting point of the polypropylene plastic polymers. The optimized melting point of the polypropylene is ranged from 160 to 166 ℃ depending on the atactic materials and crystalline (Rychlý et al. 2011).

Additional peaks in the derivative curve from 300 to 450 °C for a medical face mask are due to the deformational or melt agglomerate transition phase formed in the polypropylene plastic polymers (Majewsky et al. 2016). In this study, the medical face masks are identified as a polypropylene type of plastic polymer, and the medical hand-on gloves are identified as nitrile rubber types of plastic polymers. Thus, it can be concluded that the identified medical plastics can be recycled into fuel energy due to they are thermoplastic polymers having high oil content.

### Pyrolysis products

**Fig. 4:**
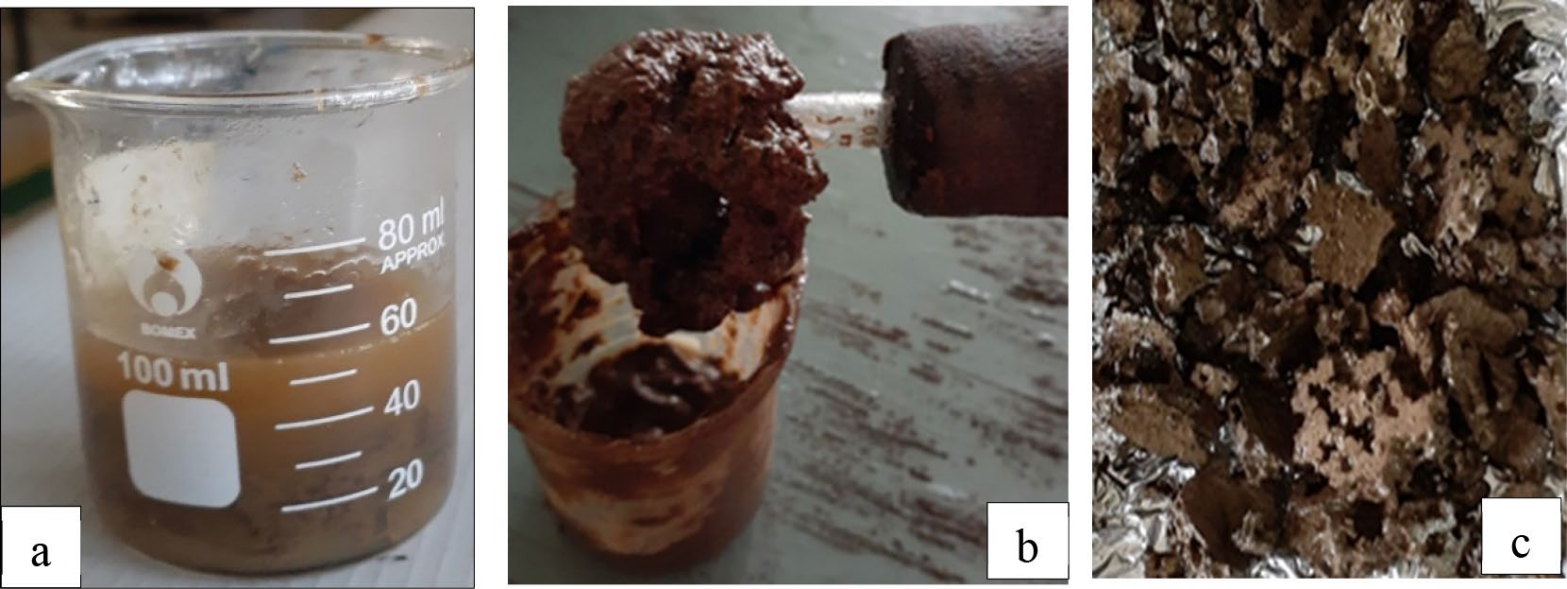
Pyrolysis products (**a**), **b** Bio-crude oil, **c** Char

The primary products from a mixed face mask and surgical gloves pyrolysis was liquid pyrolysis oil and solid char shown in Fig. 4.

As seen in Fig. 4a, tar (liquid oil and wax) and char were obtained at a temperature of 400℃ for 1 h of operation. The jelly-like liquid in Fig. 4b is produced from the heavy aromatic compound found in gloves. From total feed samples weight, 225 grams of crude oil and 30 gm of plastic residue (char) were obtained at the end of the experiment. Conferring to the experiment outcome, the recovery rate is estimated as 75 wt.%, liquid, 10 wt.% char, and the rest were released as non-condensable gases other than bio-crude oil. The present study product yields are in line with literature report the of pyrolysis of plastic wastes where 75–80% liquid oil and waxes have been produced at temperatures ranges of 500–650 ℃ (Mastral et al. 2002; Fakhrhoseini and Dastanian 2013). Furthermore, the yield was also in the range of thermal pyrolysis process common municipal plastic waste. According to the review reports by (Abnisa and Daud 2014), up to 80% of liquid oil can be obtained through pyrolysis of plastic waste around 500 ℃. Similarly (Ahmad et al. 2014) achieved 98.7%w/w liquid, 69.8%w/w gas, 28.8%w/w, and residue 1.34% w/w from pyrolysis of polypropylene at 400 ℃. (Sakata et al. 1999) also, investigate the pyrolysis of PP at a temperature of 380℃ and found 80.1 w/w% liquid yields, 6.6 wt%t gas yield, and 13.3 wt.% solid residues. FakhrHoseini and Dastanian 2013 reported 82.12 wt% liquid was found from pyrolysis of PP at 500℃. (Demirbas 2004) also revealed that pyrolysis of PP yields 48.8 wt% liquid, 49.6 wt% gas, and 1.6 wt% char at a high temperature of 740℃. In addition, plastics are essentially polymers with major constituting of carbon and hydrogen atoms (Al-Salem et al. 2017). Particularly, PE and PP have carbon content ranging from 85.5–86.1% (Sørum et al. 2005; Encinar and González 2008). Such a high carbon content present in a facemask (PP) makes pyrolysis a favorable treatment to re-use for fuel. Whereas hand-on gloves (PVC) have high aromatic content (Al-Salem et al. 2017). Hence, PVC plastics pyrolysis yields high portions of aromatics. With this regard, the fuel recovery from the PPE wastes is reasonable for reuse and in good agreement with the previous studies. However, the yield and the product composition of pyrolyzed plastic depend upon the residence time and waste polymer type of plastic waste (Miskolczi et al. 2004). Indeed, the crude oil has a similar calorific value as diesel or gasoline up on up upgrading through fractionation state (Erdogan 2020).

## Potential applications of pyrolysis products and implication for energy options

A plastic waste recovery against pollution during the pandemic for sustainable measures desires the plastic-type waste specification for action and plans (You et al. 2020). As confirmed by the FTIR, both face mask and surgical gloves are polypropylene and Nitrite rubber (PVC) thermoplastic polymers. This kind of polymer can be converted efficiently to value-added biofuels through thermal treatment (Jain et al. 2020). But, the researchers argued that PPE conversion into biofuels is not an effective environmental solution to manage plastic waste generated by the pandemic (Patrício Silva et al. 2020). Rather scaling up in innovation for sustainable and green plastics solutions, and developing dynamic and responsive plastic waste management systems should be an immediate action during and post-pandemic. Consequently, the need of rethinking and redesigning plastics (i.e., development of eco-friendly and bio-based solutions at an affordable price), along with the improvement of recycling streams to ensure proper end-of-life for those products during pandemic scenarios, should be at the highest priority. Reusable alternatives (such as for PPE) should be produced and financially incentivized at the industrial sector level (Patrício Silva et al. 2020). In addition, infectious disease institutes reported that coronaviruses can stay on commonly recycled materials for up to a few days, particularly lasts longer on plastic than other recycling materials during the pandemic aggravates the risk of contamination (Kaufman and Chasan 2020). However, the team of Indian scientists who conducted the study has found that a simple chemical process is sufficient to convert the plastic material present in PPE kits into biofuel and reported as it can also offer a long-term energy source(Jain et al. 2020); (Energy World 2020). Recently, another alternative waste to energy sustainable options has been reported by a group of scientists from the Swansea University with the led of Dr Moritz Kuehnel. The investigation is about hydrogen fuel production from PPE via photoreforming using sunlight, and they said it is cost-effective and affordable for developing countries, even. It involves the breakdown of PPE into hydrogen using a photocatalyst by absorbing light and converting it into energy to promote chemical reactions. This leads to the breakdown of plastic waste and the conversion of water into hydrogen (Steffan  2020).

Furthermore, evidence has shown that recycling of waste plastic and their mixtures have been used for the fuel production process at the medium heat temperature range from 200 to 420 ºC (Sarker et al. 2012). Moreover, it is evident from the studies of Miandial et al. that the liquid oil synthesized from the catalytic thermal treatment of plastic polymers ( PS, PE, PP, and PET) in particular or a mixture of the different proportion has a high quantity of range of aromatic compounds that are found in petroleum fuel products (Miandad et al. 2019). The liquid oil from the pyrolysis of these plastic wastes has a high heating value (HHV) of 41.7–44.2 MJ/kg, near to the conventional diesel (Miandad et al. 2019). Accordingly, the pyrolysis oil obtained from several plastic wastes has the prospective to be recycled as a substitute for conventional energy sources. According to (Lee et al. 2015) and (Rehan et al. 2016), electricity generation from pyrolysis liquid oil is attained in a diesel engine. (Saptoadi and Pratama 2015) effectively utilized Pyrolytic liquid oil as a substitute in a kerosene stove. Besides, the aromatic compounds can be used as a resource for polymerization in many chemical industries (Sarker et al. 2012; Shah and Jan 2015). In addition, several scholars used the produced liquid oil in transport sectors as fuel after mixing with commercial diesel at different ratios. Several investigations were also performed to discover the potential of pyrolysis liquid oil from the perspective of locomotive performance and automobile exhaust gas emission. (Nileshkumar et al. 2015) and (Lee et al. 2015) stated that a 20:80% mixing ratio of pyrolytic oil and conventional diesel, respectively, offered analogous engine performance effects than conventional diesel.

The remaining char could be easily utilized as a fuel or a raw material for other petrochemicals (Kabakcı and Hacıbektaşoğlu 2017). The pyrolysis solid residue is composed of condensed organic residues and the inorganic phases, with an average high heating value of 28.5–29 MJ/kg (Kabakcı and Hacıbektaşoğlu 2017). The char can also be activated via steam and thermal activation for methylene blue dye adsorption from wastewater (López et al. 2011) (Bernardo 2011). Similarly, the char is also used for the synthesis of a novel carbon-metal adsorbent for Congo red in wastewater. Furthermore, the char can be used as a feedstock for the synthesis of activated carbon (Bernardo 2011) (Miandad et al. 2016).

The toxic by-products during pyrolysis can be controllable by adopting catalysts during the process and altering the operational parameters (heating rate, temperature, duration, etc.). Pyrolysis of plastic in a thermal closed reactor under an inert environment and/or CO_2_ also prevents toxic by-products formed through carbon rearrangement (Jung et al. 2021). Particularly, carbonaceous materials made for PPE can turn into three phases of pyrogenic products, including syngas (H_2_, CH_4,_ and CO), condensable gaseous/liquid hydrocarbons, and solid residue (char) in single or multi-stage pyrolysis over catalyst. Moreover, toxic by-products could be minimized in the thermal degradation of plastic in presence of a catalyst. This process improves the production of a large quantity of liquid oil and condensable hydrocarbon due to its capability for dehydrogenation potentially reduced toxic by-product with technical completeness in terms of air pollution controls.

## Impacts of Covid-19 plastics on the environment

Following the increase of plastics waste and alterations in waste management plans, many reports tried to estimate their environmental footprint considering different scenarios. For example, the (Hub 2020) studied a life cycle assessment on UK-wide adopted faces. The study showed that the use of reusable masks considerably lessens the extent of waste by 95%, followed by reusable face cover with one-use filters (60%). Reusable face with no filters that were washed by machine had the overall least impact on climate change (< 2.00E + 008 Kg CO_2_-eq).

On the Other hand, single-use face shield and reusable face shield with one-use filters had the highest role to climate change (∼1.47E + 009 and 1.50E + 009; respectively Kg CO_2_-eq.). Therefore, the deployments of disposable masks worsen climate change by 10 folds than reusable masks.

Though there is no current valuation for gloves, the previous investigation has shown the use and mass-scale production of the glove may be a threat to the environment. For instance, synthetic rubber gloves manufactured in Malaysia utilized 10.0413 MJ of energy per production per kg with impacts extremely reliant on energy production (Hub 2020). Meanwhile, in Thailand, the overall carbon footprint release is estimated to around 42 kg CO_2_-eq per 200 pieces of rubber glove (Usubharatana and Phungrassami 2018). Considering the expected suggested regular monthly use of 65 billion gloves globally (Prata et al. 2020), and the earlier projected carbon footprint release by (Usubharatana and Phungrassami 2018), results in the release of 1.44 × 10E + 010 Kg CO_2_-Eq. kg (14 Mt CO_2_-eq.). The deployment and choice of SUP, principally plastic bags have been in doubt over the paper and cotton in the COVID-19 period. With this scenario, the reusable options of PPE kits are the foremost step to decrease the global warming potential that comes from the use of single-use plastic and PPE (Hub 2020). Furthermore, incineration and landfilling disposal of COVID-19 pandemic plastic waste worsen the air quality in moderate- to continuing periods (Prata et al. 2020). Generation CO_2_ and CH_4_ are released in significant amounts during plastic waste decomposition in landfills or during the burning of plastics waste (Prata et al. 2020). For instance, in the UK, incineration results in 0.179 tons of CO_2_eq carbon footprint per ton of MSW while that landfilling results in 0.395 tons of CO_2_ Eq. per ton of MSW (Jeswani et al. 2013).

## Conclusion, Perspectives, and Recommendations for Future work

The rapid increase in plastic production and new plastic wastes addition due to pandemics to the environment aggravates the ecosystem threat for the forthcoming years. Particularly, the word plastic now appears to be denoted by the phrase "enemy of the environment". But, plastic in itself is not an enemy when used and disposed of properly. The plastic waste problem is neither inherent to its natural surroundings nor a problem in itself. The concern of plastic waste is our approach towards it. Meaning, it is part of the production process of various products required for social progress and welfare. The challenge is what we do with such plastic wastes. However, currently, there is huge production and utilization of PPE kits to save life lead to medical waste disposal a big concern. Particularly, the demand for the face shield and gloves are not declined as expected in the post-pandemic period up to 2025. Besides, the world is in economic crisis and ecological imbalance enforced by pandemic aiming to fight COVID-19. Thus, to eradicate the environmental threat of plastic waste particularly disposable medical waste, formulating recycling, and /or reusing alternative options as sustainable management is required attention. Some of the solutions could be to redesign the biodegradable and reusable PPE. For instance, the Vietnamese Company, shoes, and Canada's University of British Columbia BioProducts Institute produced a biodegradable face mask. While the Edinburgh study suggests cloth masks to reduce disposable PPE. Beyond the use of biodegradable and reusable PPE, recycling plastic wastes should be sought attention to preserve the sustainability of the management strategy. Owing to the thermoplastic nature of PPE kits, the authors proposed thermal degradation as an effective means of recycling. The pyrolysis experimental investigation of PPE plastics wastes converted more than 75% of waste to bio-crude oil (tar). Thus, the suggested method of recycling PPE via pyrolysis is an indicative measure to alleviate the disposal problem of the single-use medical wastes. Therefore, the reprocessing of PPE to value-added products along with proper handling eases disposal problems and provides energy sources at the same time. Thus, the contests of PPE waste and the estimated rise in energy demand could be tackled by the production of petroleum fuel from such PPE kits. Even though these medical waste disposals will be significantly reduced during the post-pandemic, the proposed strategy could be applied similarly to other plastics that existed in solid waste streams. It has been noted that the quality and yield of pyrolysis products are highly dependent on temperature and the presence of a catalyst. So the work needs further investigation using a variety of catalysts over a range of temperatures (300 – 500℃) to enhance the pyrolysis process and efficiency. In addition, crude oil obtained also needs further upgrading and GC-MS accurate quantitative analysis of chemical components of oil would be required. On top of that, a detailed technical, economic and environmental impact assessment of on potentials and challenges of pyrolysis of PPE waste to fuel using life-cycle assessment (LCA) should be conducted for strategies and policymakers.

## Data Availability

All data generated or analyzed during this study are included in the body of the manuscript.

## Abbreviations

TGA: Thermogravimetric analysis
DTA: Differential thermogravimetric analysis
FTIR: Fourier transform infrared spectroscopy
PVC: Polyvinyl chloride
PPE: Personal protective equipment
SUPs: Single-use plastics
